# Supplementary material for: Inhibition of Heme Oxygenase-1 Activity Enhances Wilms Tumor-1-Specific T-Cell Responses in Cancer Immunotherapy
Source: Int J Mol Sci. 2019 Jan 23;20(3):482. doi: 10.3390/ijms20030482 (PMC6387130; doi:10.3390/ijms20030482)
Supplement: Supplementary file 1 [file ijms-20-00482-s001.pdf]

Inhibition of heme oxygenase-1 activity enhances Wilms tumor-1-specific T-cell responses in cancer immunotherapy

Supplementary figures

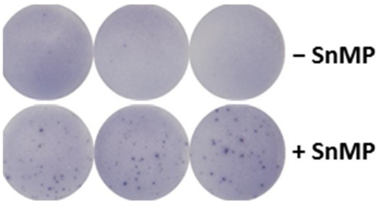

Supplementary figure S1: Representative image of an IFN-γ ELISpot analysis. Isolated PBMCs of a donor were stimulated with ppWT1 with (*bottom*) or without SnMP (*top*). Each condition was performed in triplicates. Development of the ELISpot plate was done on the next day.

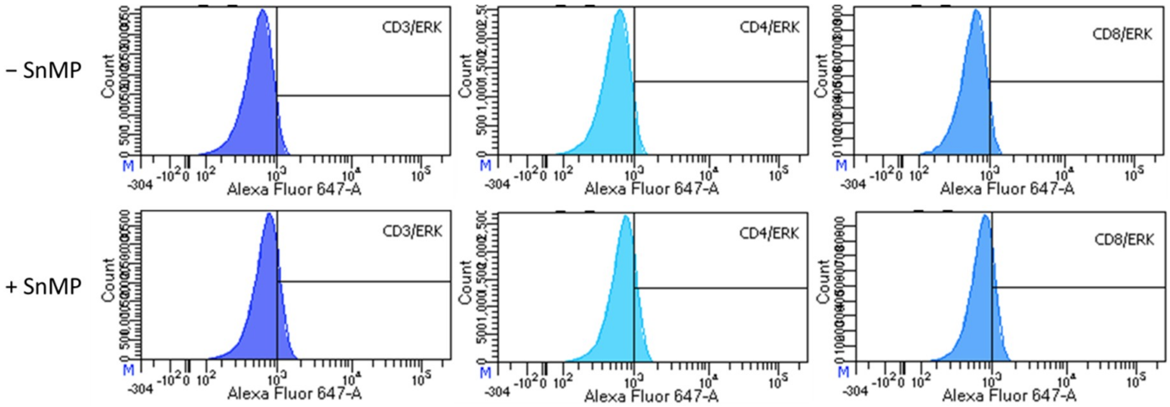

Supplementary figure S2. FACS analysis of pERK1/2. ppWT1-stimulated $CD3^+$, $CD4^+$ and $CD8^+$ cells were analyzed for their expression of pERK1/2 in the presence or absence of SnMP. Illustrated are the data of one representative donor analyzed by flow cytometry.

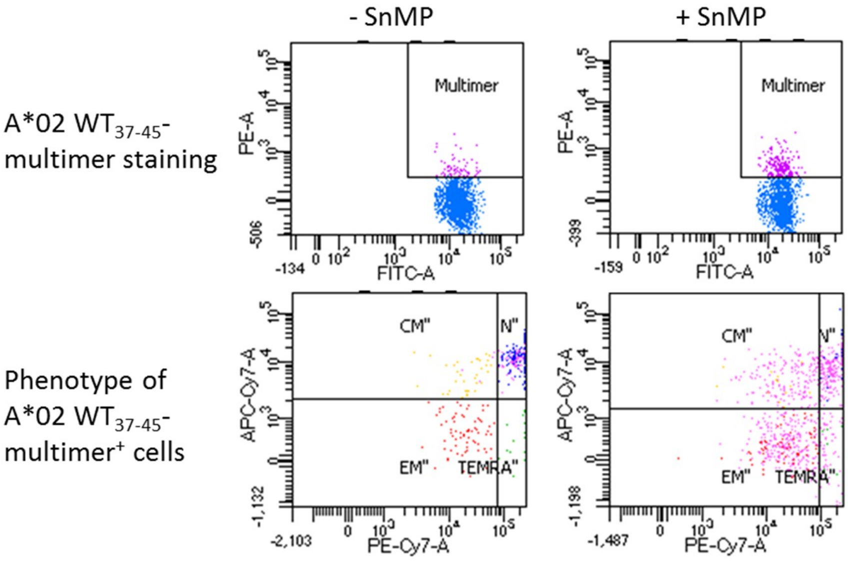

Supplementary figure S3. FACS analysis of $CD8^+$ T cells labeled with the A*02 $WT_{37\text{-}45}$-restricted multimer. Gating of the $multimer^+$ cells with or without stimulation of SnMP is shown on top and determination of the corresponding phenotype of the cells is shown in the bottom.
